# Supplementary material for: Efficacy and safety of rifaximin in preventing hepatic encephalopathy: A systematic review and meta-analysis
Source: PLoS One. 2025 May 16;20(5):e0323359. doi: 10.1371/journal.pone.0323359 (PMC12083811; doi:10.1371/journal.pone.0323359)
Supplement: S3 File — Fig S1 Cochrane Risk of Bias Assessment. Risk of bias graph (A); Risk of bias summary (B). 12 RCTs described the random sequence generation and manifested a low risk of attrition, reporting or other biases, 6 RCTs described the allocation concealment, and 5 RCTs described the blinding of participants and personnel as low risk. Fig S2 The forest plot of the effect of RFX treatment on all-cause mortality. No statistical difference in mortality risk between both overall and subgroup analyses. Fig S3 The forest plot of the effect of rifaximin treatment on blood ammonia and hospitalization rate. Blood ammonia levels after RFX treatment were mild higher than in the control group (including NADs, nitazoxanide, and placebo) (A), while more beneficial in lowering the hospitalization rate compared with the control group (B). Fig S4 Funnel diagram of RFX for prevention of HE. Funnel plot was asymmetrical that suggesting the possible publication bias. (DOCX) [file pone.0323359.s003.docx]

**Supplementary Figure S1-4**

**Contents**

*Fig S1 Cochrane Risk of Bias Assessment*

*Fig S2 The forest plot of the effect of RFX treatment on all-cause mortality*

*Fig S3 The forest plot of the effect of rifaximin treatment on blood ammonia and hospitalization rate*

*Fig S4 Funnel diagram of RFX for prevention of HE*


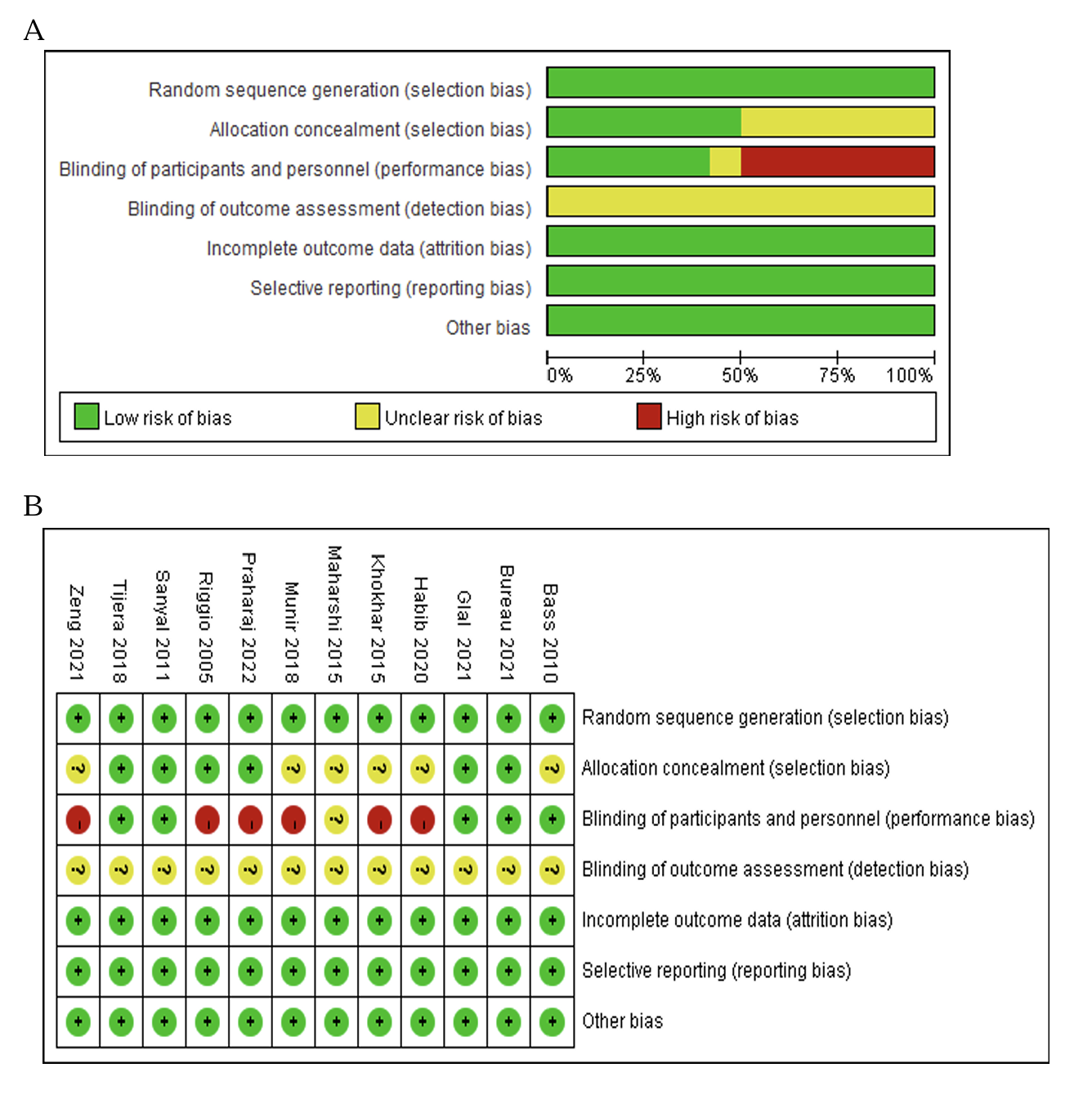


**Fig S1** **Cochrane Risk of Bias Assessment**. Risk of bias graph (A); Risk of bias summary (B)**.** 12 RCTs described the random sequence generation and manifested a low risk of attrition, reporting or other biases, 6 RCTs described the allocation concealment, and 5 RCTs described the blinding of participants and personnel as low risk.


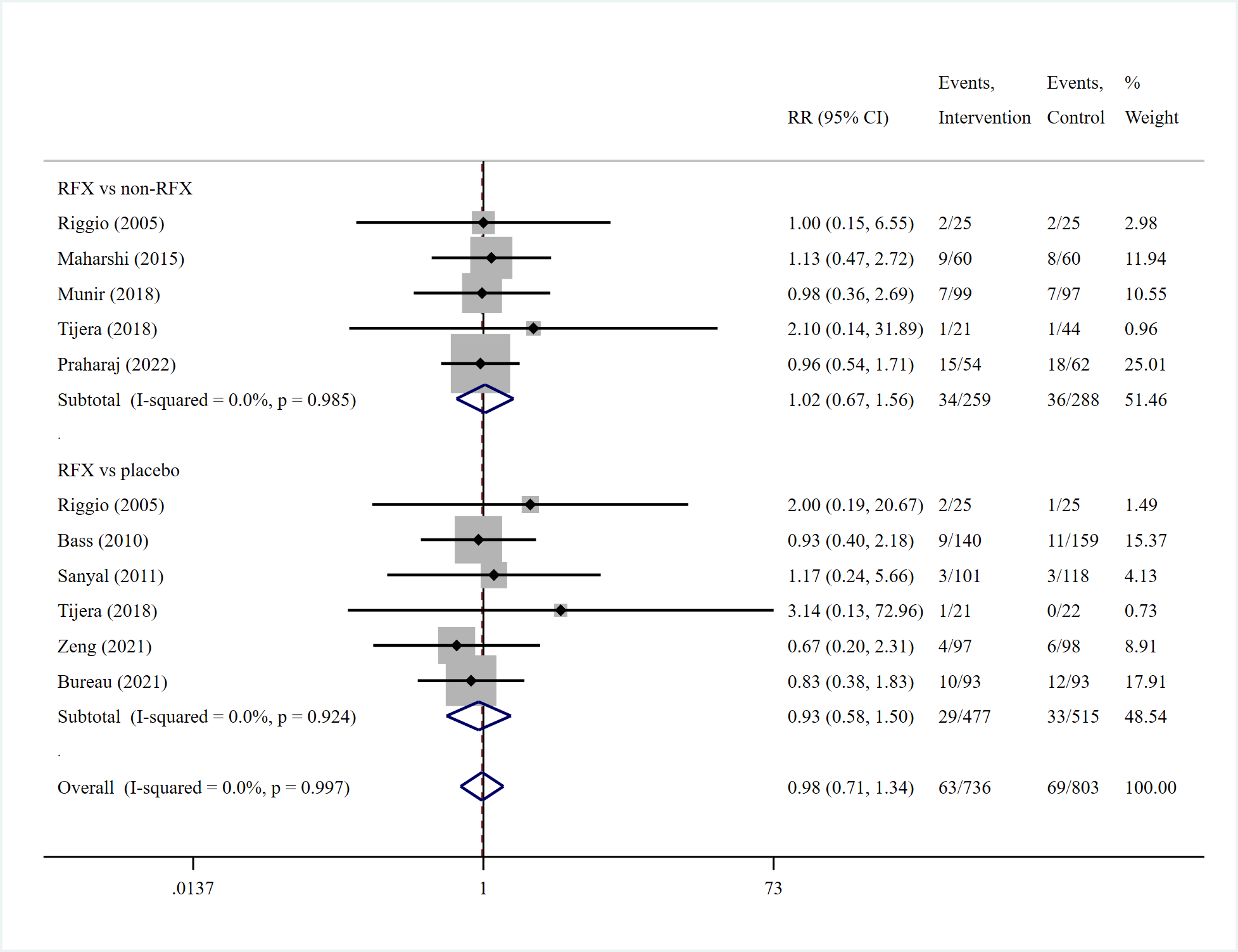


**Fig S2** **The forest plot of the effect of RFX treatment on all-cause mortality.** No statistical difference in mortality risk between both overall and subgroup analyses.


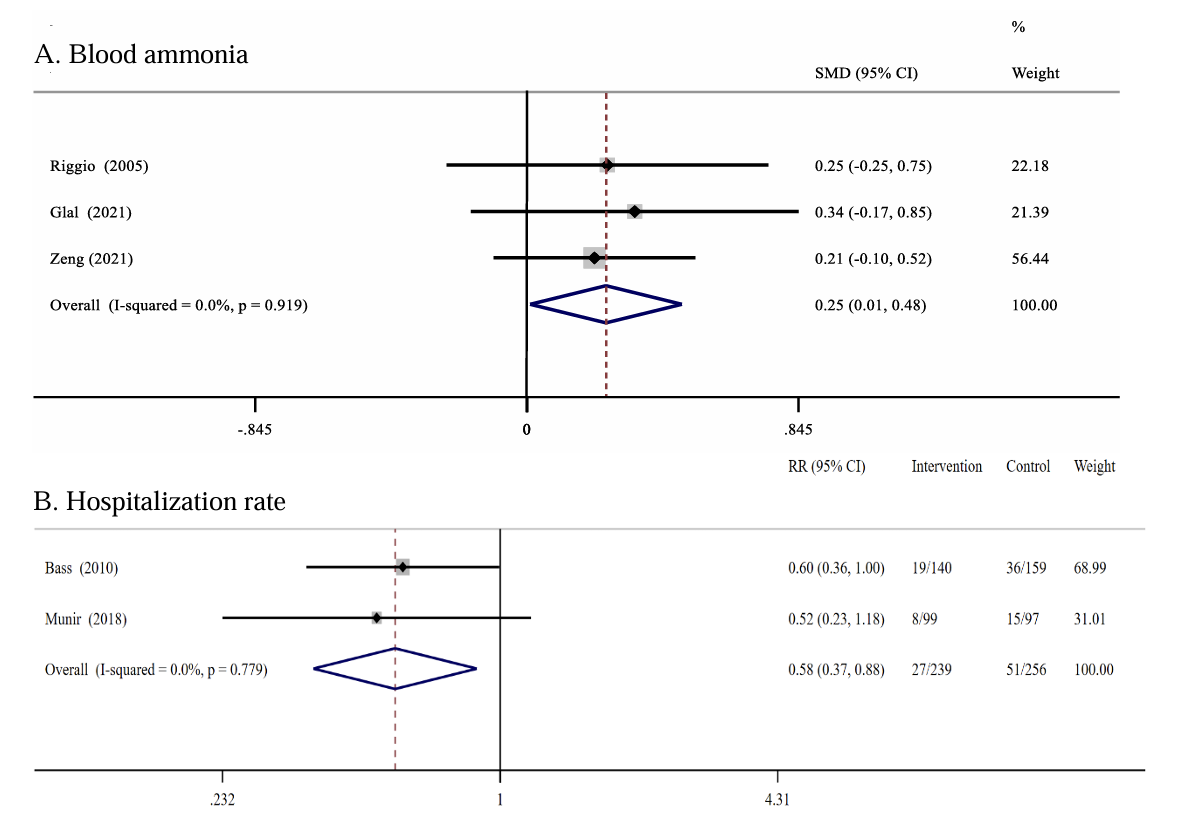


**Fig S3** **The forest plot of the effect of rifaximin treatment on blood ammonia and hospitalization rate****.** Blood ammonia levels after RFX treatment were mild higher than in the control group (including NADs, nitazoxanide, and placebo) (A), while more beneficial in lowering the hospitalization rate compared with the control group (B).


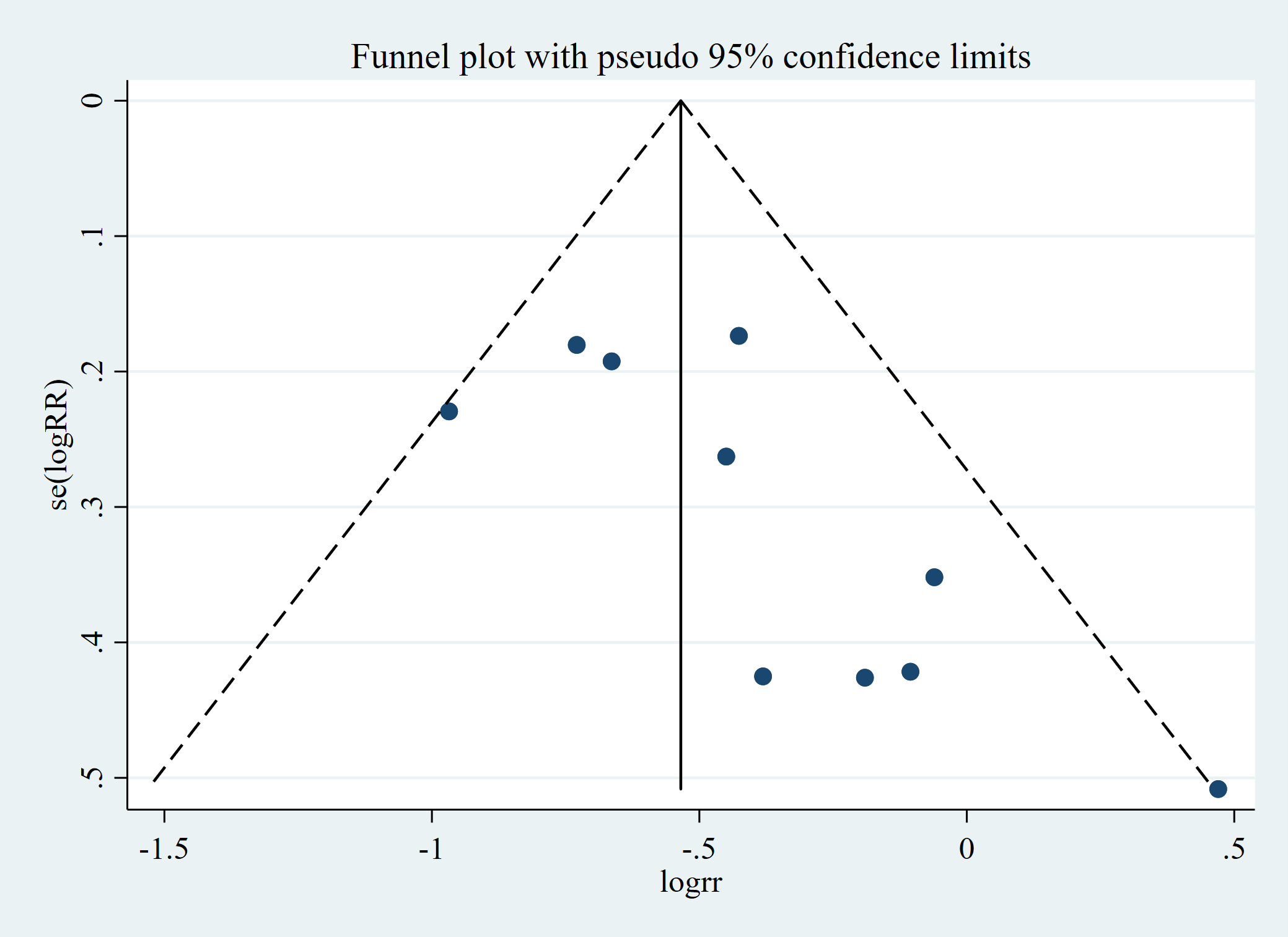


**Fig S4** **Funnel diagram of RFX for prevention of HE.** Funnel plot was asymmetrical that suggesting the possible publication bias.

Article Title: **Efficacy and safety of** **Rifaximin in preventing hepatic encephalopathy: a Systematic Review and Meta-Analysis**

Corresponding Author Name: Yadong Wang, MD PhD

Department of Infectious Diseases, Hebei Medical University Third Hospital, No. 139, Ziqiang Road, Shijiazhuang, Hebei 050051, China.

Tel: +86-311-66776831

+86-18533112392

Email: wangyadong@hebmu.edu.cn

ORCID: 0000-0003-0140-0674
