# Supplementary material for: Efficacy and safety of rifaximin in preventing hepatic encephalopathy: A systematic review and meta-analysis
Source: PLoS One. 2025 May 16;20(5):e0323359. doi: 10.1371/journal.pone.0323359 (PMC12083811; doi:10.1371/journal.pone.0323359)
Supplement: S4 File — Table S1 The basic information of selected literature. Table S2 The incidence of adverse events during RFX treatment. (DOCX) [file pone.0323359.s004.docx]

**Supplementary Table S1-2**

**Contents**

*Table S1 The basic information of selected literature*

*Table S2 The incidence of adverse events during RFX treatment*

| **Table S1 The basic information of selected literature** | | | | | | | | |
| --- | --- | --- | --- | --- | --- | --- | --- | --- |
| Study | Study design | Participants | Sample Size | | Interventions | | Treatment duration | Outcomes |
|  |  |  | RFX | Control | RFX | Control |  |  |
| Riggio 2005^14^ | Double-blind, RCT | Cirrhotic patients submitted to TIPS | 25 | 50 | RFX 400mg tid | Lactitol 60mL/d, placebo | 30days | Incidence of HE, blood ammonia level, all-cause mortality, etc. |
| Bass  2010^13^ | Multicentre, double  -blind, RCT | Cirrhotic patients with recurrent HE in remission | 140 | 159 | RFX 550mg bid | Placebo | 6moths | Incidence of HE, all-cause mortality, hospitalization rate, adverse events, etc. |
| Sanyal 2011^15^ | Double-blind, RCT | Cirrhotic patients with recurrent HE in remission | 101 | 118 | RFX 550mg bid | Placebo | 6moths | Incidence of HE, all-cause mortality, etc. |
| Khokhar 2015^16^ | RCT | Cirrhosis with a history of previous HE episodes | 128 | 178 | RFX 550mg bid | RFX 550mg bid | 6moths | Incidence of HE, etc. |
| Maharshi 2015^17^ | RCT | Cirrhotics with acute variceal bleeding | 60 | 60 | RFX 400mg bid | Lactulose 120mL/d | 5days | Incidence of HE, all-cause mortality, adverse events, etc. |
| Munir  2018^10^ | RCT | Cirrhotic patients with recurrent HE in remission | 99 | 97 | RFX 550mg bid | Lactulose 30-120mL/d | 6moths | Incidence of HE, all-cause mortality, hospitalization rate, adverse events, etc. |
| Tijera  2018^18^ | Double-blind, RCT | Cirrhotics with variceal bleeding | 21 | 66 | RFX 400mg tid | Lactulose 10-90mL/d, LOLA10g/d | 7days | Incidence of HE, all-cause mortality, adverse events, etc. |
| Habib  2020^19^ | RCT | Cirrhosis | 40 | 40 | RFX 200mg bid | RFX 550mg bid | 6moths | Incidence of HE, *etc*. |
| Bureau  2021^20^ | Multicentre, double-blind, RCT | Cirrhotic patients submitted to TIPS | 93 | 93 | RFX 600mg bid | Placebo | 6moths | Incidence of HE, all-cause mortality, *etc*. |
| Glal  2021^21^ | Double-blind, RCT | Cirrhosis with a history of previous HE episodes | 30 | 30 | RFX 550mg bid | Nitazoxanide 500mg bid | 6moths | Incidence of HE, blood ammonia level, adverse events, *etc*. |
| Zeng  2021^22^ | RCT | Decompensated cirrhosis | 97 | 98 | RFX 400mg tid | Placebo | 6moths | Incidence of HE, all-cause mortality, blood ammonia level, adverse events, *etc*. |
| Praharaj 2022^23^ | RCT | Cirrhosis with ascites | 54 | 62 | RFX 550mg bid | Norfloxacin 400mg qd | 6moths | Incidence of HE, all-cause mortality, *etc*. |

Abbreviations: TIPS, Transjugular intrahepatic portosystemic shunt; RFX, Rifaximin; HE, Hepatic encephalopathy; LOLA, L-ornithine-L-aspartate

**Table S2** **The incidence of adverse events during RFX treatment**

| Adverse events | Sample Size | | Heterogeneity | | RR (95%CI) | *P* |
| --- | --- | --- | --- | --- | --- | --- |
|  | RFX | Control | *P* | I^2^ |  |  |
| Dizziness or headache | 267 | 287 | 0.920 | 0.0% | 1.16（0.79，1.70） | 0.461 |
| Abdominal pain | 299 | 316 | 0.394 | 0.0% | 1.35（0.68，2.68） | 0.396 |
| Diarrhea | 348 | 413 | 0.025 | 64.1% | 0.48（0.14，1.63） | 0.236 |
| RFX vs NADs | 81 | 82 | 0.872 | 0.0% | 0.04（0.00，0.25） | 0.001 |
| RFX vs placebo | 258 | 279 | 0.372 | 0.0% | 0.70（0.41，1.20） | 0.194 |
| Nausea or vomiting | 320 | 382 | 0.293 | 19.5% | 1.14（0.76，1.71） | 0.515 |
| RFX vs non-RFX | 180 | 201 | 0.566 | 0.0% | 2.98 (0.82，10.87） | 0.098 |
| RFX vs placebo | 161 | 181 | 0.469 | 0.0% | 1.01（0.66，1.54） | 0.981 |
| Bloating or abdominal discomfort | 221 | 285 | 0.038 | 69.5% | 0.27（0.03，2.46） | 0.246 |
| Constipation | 267 | 287 | 0.304 | 16.0% | 0.95（0.47，1.92） | 0.881 |
| Rash | 267 | 287 | 0.717 | 0.0% | 1.19（0.52，2.74） | 0.680 |

Article Title: **Efficacy and safety of** **Rifaximin in preventing hepatic encephalopathy: a Systematic Review and Meta-Analysis**

Corresponding Author Name: Yadong Wang, MD PhD

Department of Infectious Diseases, Hebei Medical University Third Hospital, No. 139, Ziqiang Road, Shijiazhuang, Hebei 050051, China.

Tel: +86-311-66776831

+86-18533112392

Email: wangyadong@hebmu.edu.cn

ORCID: 0000-0003-0140-0674
