# Supplementary material for: Efficacy and safety of rifaximin in preventing hepatic encephalopathy: A systematic review and meta-analysis
Source: PLoS One. 2025 May 16;20(5):e0323359. doi: 10.1371/journal.pone.0323359 (PMC12083811; doi:10.1371/journal.pone.0323359)
Supplement: Attachment — Submitted filename: Reply letter to editor.docx [file pone.0323359.s006.docx]

**Reply letter to editor:**

We've checked your submission and before we can proceed, we need you to address the following issues:

1. Please note that funding information should not appear in any section or other areas of your manuscript. We will only publish funding information present in the Funding Statement section of the online submission form. Please remove any funding-related text from the manuscript.

**Reply:** As requested by your editorial office, we have deleted funding information in our manuscript.

2. Thank you for stating the following financial disclosure:

"This study was supported by grants from the Natural Science Foundation of Hebei Province of China (H2023206042)."

If this statement is not correct you must amend it as needed. Please include this amended Role of Funder statement in your cover letter; we will change the online submission form on your behalf.

**Reply:** Thank you to the editor for your careful reminders and suggestions, as you said: The funders have no role in study design, data collection and analysis, decision to publish, or preparation of the manuscript. However, this Foundations will be used to pay Open Access costs in the later stage. If labeling is indeed not required, it can be removed completely according to your recommendations.

3. Thank you for updating your data availability statement. You note that your data are available within the Supporting Information files, but no such files have been included with your submission. At this time we ask that you please upload your minimal data set as a Supporting Information file, or to a public repository such as Figshare or Dryad.

Please also ensure that when you upload your file you include separate captions for your supplementary files at the end of your manuscript.

As soon as you confirm the location of the data underlying your findings, we will be able to proceed with the review of your submission.

**Reply:** According to our data availability statement, in addition to the manuscript, we uploaded *Table S1-2*, *Figure S1-4*, as well as links to "*Table includes all literature and rationale for exclusion* and *PRISMA checklist*". These were obtained in the Manuscript and Supplementary Files.

4. Please include a separate legend for each figure in your manuscript.

**Reply:** I am very sorry that it was not completed in time. In the latest revised manuscript, we have supplemented the figure legend of Fig1-4 and S1-S4. Please review it again.

5. Thank you for addressing most of our recent requests. However, the following item still appears to be outstanding:

As required by our policy on Data Availability, please ensure your manuscript or supplementary information includes the following:

A numbered table of all studies identified in the literature search, including those that were excluded from the analyses.

a. For every excluded study, the table should list the reason(s) for exclusion.

b. If any of the included studies are unpublished, include a link (URL) to the primary source or detailed information about how the content can be accessed.

You have included the 12 articles you included in your final analysis, but the total number of studies you identified for screening was 1906. Please can you add a table that includes all 1906 papers.

Please could you upload this table as a "supporting information" file or provide a link to a repository containing the file?

**Reply:** Thanks to the editors' careful review and sincere suggestions, we have uploaded all retrieved articles as attachments in the form of EXCEL tables (Table includes all literature and exclusion grounds), which include a list of all primary screened articles, as well as a description of the reasons for exclusion.

Thank you very much!

Your sincerely

Yadong Wang *et al.*
